# VISA - Vector Integration Site Analysis server: a web-based server to rapidly identify retroviral integration sites from next-generation sequencing

**DOI:** 10.1186/s12859-015-0653-6

**Published:** 2015-07-07

**Authors:** Jonah D. Hocum, Logan R. Battrell, Ryan Maynard, Jennifer E. Adair, Brian C. Beard, David J. Rawlings, Hans-Peter Kiem, Daniel G. Miller, Grant D. Trobridge

**Affiliations:** Department of Pharmaceutical Sciences, Washington State University, Spokane, WA 99210 USA; Clinical Research Division, Fred Hutchinson Cancer Research Center, Seattle, WA 98109 USA; Department of Pediatrics, University of Washington, Seattle, WA 98195 USA; School of Molecular Biosciences, Washington State University, Pullman, WA 99164 USA

**Keywords:** Bioinformatics, Gene therapy, Retroviral vectors, Next-generation sequencing, Retroviral mutagenesis

## Abstract

**Background:**

Analyzing the integration profile of retroviral vectors is a vital step in determining their potential genotoxic effects and developing safer vectors for therapeutic use. Identifying retroviral vector integration sites is also important for retroviral mutagenesis screens.

**Results:**

We developed VISA, a vector integration site analysis server, to analyze next-generation sequencing data for retroviral vector integration sites. Sequence reads that contain a provirus are mapped to the human genome, sequence reads that cannot be localized to a unique location in the genome are filtered out, and then unique retroviral vector integration sites are determined based on the alignment scores of the remaining sequence reads.

**Conclusions:**

VISA offers a simple web interface to upload sequence files and results are returned in a concise tabular format to allow rapid analysis of retroviral vector integration sites.

**Electronic supplementary material:**

The online version of this article (doi:10.1186/s12859-015-0653-6) contains supplementary material, which is available to authorized users.

## Background

Retroviral vector mediated gene therapy has enormous potential for treating genetic disorders [1] and infectious diseases [2]. Unfortunately, vector integration can lead to the dysregulation of nearby genes, commonly referred to as genotoxicity [3]. Prime examples of the effects of genotoxicity include two X-linked severe combined immunodeficiency (SCID-X1) patients who were treated with a gammaretroviral vector in a clinical trial and developed lymphoproliferative leukemias a few years later [4]. It was discovered that transduced cells from the patients contained integration sites near the *LMO2* gene and that there was aberrant expression of *LMO2*. Taken together, this suggested that vector-mediated dysregulation of *LMO2* led to clonal dominance and the development of leukemia in the patients. This study highlights the importance of evaluating the safety of retroviral vectors.

Identifying retroviral vector integration sites (RISs) is critical to assess genotoxicity in gene therapy clinical trials and to develop improved vectors in preclinical studies. Another use of RIS mapping is for retroviral mutagenesis screens. In these screens genes identified near the provirus are candidate cancer initiation or progression genes [5]. Retroviral proviruses act as molecular tags, enabling the detection of RISs via methods such as linear amplification-mediated (LAM)-PCR and other next generation sequencing (NGS) methods [6, 7]. NGS can generate millions of sequence reads and an individual RIS can be represented multiple times in NGS data, making the identification and annotation of RISs challenging. We present a Vector Integration Site Analysis (VISA) server, a tool that allows investigators with limited bioinformatics experience to rapidly analyze large NGS datasets for RISs.

## Implementation

### Identify LTR-chromosome junctions and generate query sequences

Sequencing DNA samples from retroviral vector integration studies with a long terminal repeat (LTR) primer produces sequence reads with LTR-chromosome junctions, with the LTR sequence flanking the 5′ end of the chromosome/genomic sequence. Methods such as LAM-PCR will additionally result in a linker cassette (LC) sequence flanking the 3′ end of the genomic sequence. VISA uses a Perl substring matching strategy to detect and remove these non-genomic sequences to generate the queries for alignment (see Additional file 1 section ‘Trimming non-genomic portions of the sequence reads’ for details). VISA accepts multiple sequence reads in a single FASTA formatted file as input. Each sequence is trimmed with the following steps: (1) The vector LTR sequence is searched for in the sequence read. If the LTR sequence is found, the query begins downstream of the LTR position. (2, optional) The LC sequence is searched for in the query. If the LC sequence is found, the query is truncated upstream of the LC position. (3) If the sequence read contains a valid query, the query will be truncated if 3 or more consecutive ambiguous bases , ‘Ns’, are detected to eliminate queries with poor sequence quality. (4) If the query is less than 30 bp it is eliminated, since it will be below the alignment score cutoff (see section ‘Align query sequences to the genome and filter alignments’ for details). Only sequence reads that contain an LTR-chromosome junction and result in a query that is at least 30 bp are considered for alignment. Searching for a LC sequence is optional to maximize the flexibility of VISA.

### Align query sequences to the genome and filter alignments

Query sequences are aligned to the Genome Reference Consortium Human Build 38 (hg38) and the selected vector sequence using BLAT [8]. BLAT is used with the following parameters: blat.exe chromosome_file query_file -out = blast8 -tileSize = 11 -stepSize = 5 -ooc = 11-2253.ooc output_file (see Additional file 1 for details about the generation of the ooc file). Users have the option of processing sequence reads without using the ooc file as well. Alignments with an alignment score < 60, a percent identity < 92 %, and/or that start more than 3 bp from the query start site are no longer considered for processing. For the remaining alignments, the 5 greatest scoring alignments of each query sequence are retained for further processing. These initial filtering steps are done using a MySQL database on a separate server, reducing the amount of memory needed by the application server to process each input file.

Additional filtering criteria are applied to the greatest scoring alignments of each query sequence to eliminate RISs that cannot be unequivocally aligned to the genome. The filters, applied in order, are as follows:

1. The greatest scoring alignment is to the vector sequence.
2. The second greatest scoring alignment has an alignment score > 95 % than that of the greatest scoring alignment. For lower scoring alignments, alignment scores < 100, this is reduced to 90 %.
3. The greatest scoring alignment has a percent identity < 95 %.

For query sequences that exceed the elimination criteria in quality and confidence, it is assumed that the greatest scoring alignment is the RIS for the associated sequence read and is labeled a ‘candidate RIS’. Query sequences that do not meet the criteria are filtered out and reported separately from the candidate RISs in the results.

### Identify unique retroviral vector integration sites

There can be repeated recovery of a specific RIS due to PCR amplification bias or legitimate clonal expansion after integration. Also, sequencing errors can lead to mapping of the same RIS within a few bp of the actual integration site. Both of these potential scenarios are handled by assuming candidate RISs within ±5 bp of the same chromosome location represent the same RIS. Candidate RISs are binned and sorted by chromosome locations. Then, within each 10 bp window (±5 bp) the candidate RIS with the greatest alignment score is assigned as the unique RIS. Candidate RISs that align to the same chromosome location as a unique RIS are labeled ‘repeat RIS’ and those that align within the 10 bp window of a unique RIS are labeled ‘in range RIS’. The frequency and the number of distinct bp spans of each unique RIS is reported (see Additional file 1 section ‘Identifying unique retroviral vector integration sites’ for an example and a discussion of the 10 bp window for grouping candidate RISs).

### Determine proximity of unique retroviral vector integration sites to nearby genes

Unique RISs can also be further processed to determine their proximity to nearby genes using RefSeq gene annotations and a user-provided list of genomic features. Large scale integration analyses have shown that different types of retroviral vectors have distinct integration profiles, e.g., lentiviral vectors have a strong preference to integrate within genes [9] whereas foamy viral vectors have a modest preference for transcription start sites [10]. Also, the position of the provirus relative to a gene may influence the mechanism by which the provirus can potentially dysregulate the gene. For example, enhancer activation may occur when integration occurs upstream of the gene and premature polyadenylation may occur when integration occurs within a gene. For these reasons, VISA determines the proximity of RISs to genes in 2 distinct phases: (1) VISA determines if a unique RIS is within a gene. If the unique RIS is not within a gene, its distance to the nearest gene, up to 5 kb away from either the 5′ end or the 3′ end, is determined. (2) Then VISA determines the distance of the unique RIS to the nearest transcription start site of a gene. It is possible for a provirus to be within a particular gene, but actually be in closer proximity to the transcription start site of another gene. Other relevant information, such as the percent of unique RISs within genes and the number of unique RISs within 5 kb of genes, is also reported. When determining the proximity of RISs to user-provided genomic features, VISA determines if each RIS is within a specific genomic feature or its distance to the closest genomic feature (see Additional file 1 section ‘Determining the proximity of unique RISs to a user-provided list of genomic features’ for the requirements of the genomic feature list). Pearson's chi-squared goodness of fit test is used to determine the significance of these results. The proximity of 100,000 randomly generated integration sites to the set of RefSeq genes and custom genomic features are used as the expected values in the Pearson chi-squared goodness of fit analyses.

### Generate random integration sites

VISA can also generate random integration datasets for use as a control. Randomly selected sites are extracted from the hg38 chromosome sequences. Users can specify random site lengths from 30 to 1000 bp and the number of random sites from 1 to 100,000. Randomly generated sites are then processed the same as datasets generated by NGS to impart the same biases that occur from alignment to hg38.

## Results and discussion

**Figure 1:**
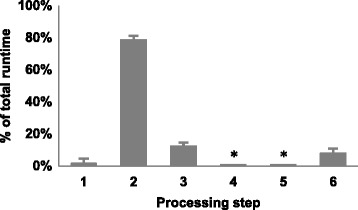
VISA requires minimal processing time relative to the alignment process. The processing steps are as follows: (1) Identify LTR-chromosome junctions and generate query sequences, (2) align query sequences to the genome using BLAT, (3) retrieve the top 5 alignments for each query sequence and filter out alignments that cannot be aligned to a unique location in the genome, (4) identify the unique RISs, (5) determine proximity of unique RISs to nearby genes, and (6) prepare the excel and CSV output files. Sequence reads containing lentiviral LTR-chromosome junctions were processed with VISA. 3 input files were used with a mean of 502,083 sequence reads per file. Standard error bars are shown. * indicates the value is less than 1 %

**Figure 2:**
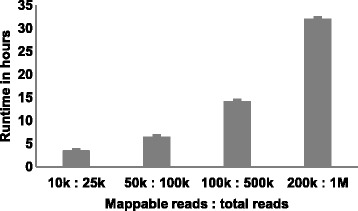
VISA is scalable with NGS. Several factors affect the amount of processing time required for an input file, including the number of reads that can be mapped to the genome and the number of files being processed in parallel. Each input file was processed 3 times. Standard error bars are shown

VISA processes sequence reads and identifies unique RISs without adding a substantial amount of time after the alignment process (Fig. 1). Memory efficiency is largely accomplished by employing a MySQL database for the initial alignment filtering. This strategy reduces the strain on the server resources while processing large NGS datasets in parallel. Binning the candidate RISs into 10 bp windows and then designating the unique RISs based on the alignment scores is a time efficient method to identify unique RISs. The compromise between time and memory efficiency allows VISA to be scalable with NGS (Fig. 2).

VISA allows investigators to analyze NGS data for RISs from human cell and tissue samples without the need to develop custom scripts. Using VISA provides a consistent method to analyze the integration profiles of retroviral vectors for preclinical and clinical studies. The frequency and span count of unique RISs and the proximity of unique RISs to genes can provide insight into both the genotoxicity of vectors and the clonality of the transduced cells. Selecting the unique RISs based on the alignment scores ensures that the sequence read with the best alignment represents each RIS. VISA has two important advantages over previously reported RIS tools [11–14]; reporting the span count in conjunction with the frequency of unique RISs, which provides a means of quality control when analyzing the clonality of transduced cells with certain PCR based methods [7], and the ability to generate random RIS datasets, that have the same biases in localization to hg38, as control datasets. VISA also is extremely flexible. The only requirement to use VISA is that the uploaded sequence file is in the FASTA format and that the appropriate vector and LTR sequence has been provided or selected via the dropdown menu. Also, the parameters discussed above used to filter the alignments and to identify unique RISs are the default and recommended settings, but these can be changed by users who find other criteria more appropriate. This flexibility means VISA can be used to analyze data from various NGS methods. VISA provides a concise and a complete version of the results from processed sequence reads. The concise version of the results contains the unique RISs and emphasizes pertinent information, such as the frequency and span counts, the alignment scores, and the proximity of unique RISs to RefSeq genes. For investigators who would like to look into why certain sequence reads did not contain a unique RIS, the complete version of the results contains the filtered alignments in separate Excel sheets. All results are provided in a tabular format as an Excel document or as compressed CSV files through a private and secure link once processing is complete. Finally, data can be distributed to users via their email, but VISA also allows anonymous usage. Example input and output files and other helpful links can be found on the VISA homepage [15].

## Conclusions

VISA is a time and memory efficient web-based tool that allows investigators to analyze large NGS datasets for RISs in a consistent manner. Results are returned in a simple format to allow rapid analysis of the integration profile and genotoxicity of retroviral vectors and mapping of RIS for retroviral mutagenesis screens.

## Availability and requirements

**Project name:** VISA (Vector Integration Site Analysis)

**Project home page:**https://visa.pharmacy.wsu.edu/bioinformatics/

**Operating systems:** Platform independent

**Programming language:** Perl, MySQL

**Other requirements:** Firefox, Chrome, IE, or Safari. JavaScript must be enabled.

**License:** None

**Any restrictions to use by non-academics:** VISA cannot be used for commercial use.

## Additional file

Additional file 1: ᅟ**Supplementary methods, results, figures, and examples.**

## Abbreviations

VISA: Vector Integration Site Analysis
SCID-X1: X-linked severe combined immunodeficiency
RIS: Retroviral vector integration site
LAM-PCR: Linear amplification-mediated PCR
NGS: Next-generation sequencing
LTR: Long terminal repeat
hg38: The Genome Reference Consortium Human build 38
