# Supplementary material for: VISA - Vector Integration Site Analysis server: a web-based server to rapidly identify retroviral integration sites from next-generation sequencing
Source: BMC Bioinformatics. 2015 Jul 7;16(1):212. doi: 10.1186/s12859-015-0653-6 (PMC4493804; doi:10.1186/s12859-015-0653-6)
Supplement: Additional file 1: — ᅟSupplementary methods, results, figures, and examples. [file 12859_2015_653_MOESM1_ESM.pdf]

Trimming non-genomic portions of the sequence reads:

VISA uses a Perl substring matching strategy to detect and remove the non-genomic sequences from the sequence reads to generate the queries for alignment. 24 bp of the LTR sequence are used to generate the 'LTR substrings' for substring matching. 3 overlapping LTR substrings are generated, each consisting of 12 bp. LTR substring 1 is the most 3′ 12 bp of the LTR sequence, LTR substring 2 is shifted 6 bp upstream from the 3′ end of the LTR sequence, and LTR substring 3 is shifted 12 bp upstream from the 3′ end of the LTR sequence. 21 bp of the linker cassette (LC) sequence are used to generate the 'LC substrings'. 3 overlapping LC substrings are generated, each consisting of 9 bp. LC substring 1 is the most 5′ 9 bp of the LC sequence, LC substring 2 is shifted 6 bp downstream from the 5′ end of the LC sequence, and LC substring 3 is shifted 12 bp downstream from the 5′ end of the LC sequence. Supplemental Figure 1 illustrates this process.

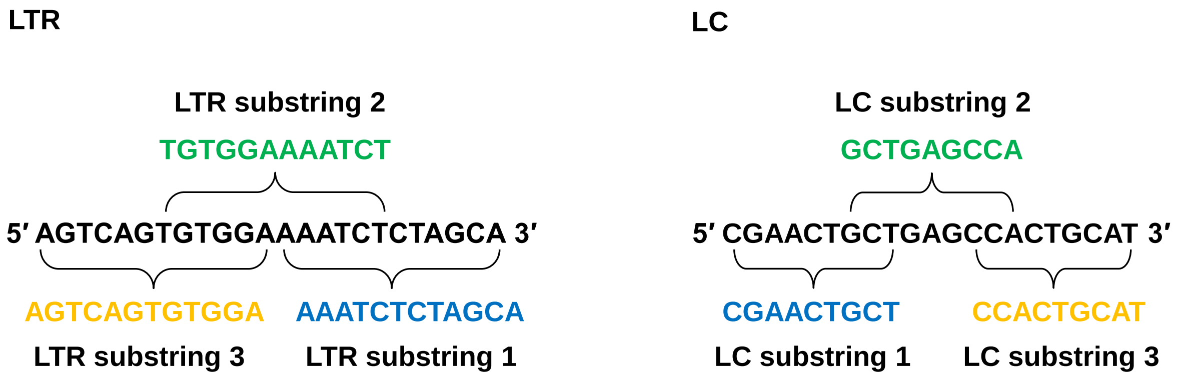

Supplemental Figure 1 LTR and linker cassette (LC) substrings. This example illustrates the process of generating the LTR and LC substrings for substring matching. A lentiviral vector LTR and a hypothetical LC are shown.

LTR substring 1 is searched for first. If LTR substring 1 is found, the query sequence begins immediately following its position and the LTR-Chromosome junction is deemed 'normal'. If a mismatch in the LTR portion of the sequence read causes LTR substring 1 not to be found, VISA then searches for LTR substring 2. If LTR substring 2 is found, the query will begin 6 bp downstream of its position to compensate for the 6 bp shift. If a mismatch in the LTR portion of the sequence read causes LTR substring 2 not to be found, VISA then searches for LTR substring 3. If LTR substring 3 is found, the query will begin 12 bp downstream of its position to compensate for the 12 bp shift. When either LTR substring 2 or LTR substring 3 are found, the LTR-Chromosome junction is deemed 'imperfect'. If a mismatch causes LTR substring 3 not to be found, the above steps are repeated using the sequence read in its reverse orientation. If none of the LTR substrings are found in either orientation, the sequence read does not contain a valid query and will not be aligned to the genome. If a LC sequence is provided, VISA then searches for the LC substrings so the query can be truncated appropriately. The same strategy is employed to detect the LC substrings. If LC substring 1 is found, the query is truncated at its position, if LC substring 2 is found, the query is truncated 6 bp upstream of its position, and if LC substring 3 is found, the query is truncated 12 bp upstream of its position. When LC substring 1 is found the linker is deemed 'normal' and when either LC substring 2 or LC substring 3 are found the linker is deemed 'imperfect'. If a LC substring is not found, or a LC sequence is not provided, the query will continue through the rest of the sequence read or until 3 ambiguous bases, 'Ns', are detected. Supplemental Figure 2 illustrates 3 examples.

Example 1: Normal LTR-Chromosome junction and normal linker

AGTAGTGTGTGCCCGTCTGTTGTGTGACTCTGGTAACTAGAGATCCCCCAGACCCTTTTAG

LTR substring 1

TCAGTGTGGAAAATCTCTAGCACATATCACTCAAGAAAATAACTAGGCTGTATTAACATTGT

LC substring 1

CATTTGGGTAATACTTTGTATGATTATTACTCCGAACTGCTGAGCCACTGCATTCAGATCC

Example 2: Imperfect LTR-Chromosome junction and imperfect linker

AGTAGTGTGTGCCCGTCTGTTGTGTGACTCTGGTAACTAGAGATCCCCCAGACCCTTTTAG

LTR substring 2 +6 bp

TCAGTGTGGAAAATCTCTATCACATATCACTCAAGAAAATAACTAGGCTGTATTAACATTGT

-12 bp LC substring 3

CATTTGGGTAATACTTTGTATGATTATTACTCCGATCTGCTCAGCCACTGCATTCAGATCC

Example 3: Normal LTR-Chromosome junction and no LC sequence provided

AGTAGTGTGTGCCCGTCTGTTGTGTGACTCTGGTAACTAGAGATCCCCCAGACCCTTTTAG

LTR substring 1

TCAGTGTGGAAAATCTCTAGCACATATCACTCAAGAAAATAACTAGGCTGTATTAACATTGT

CATTTGGGTAATACTTTGTATGATTATTACTCCGAACTGCTGAGCCACTGNATTCANNNCC

Supplemental Figure 2 Trimming LTR and linker cassette (LC) sequences from input sequence reads. Queries are underlined. Mismatches in the LTR and LC regions and ambiguous bases, ‘Ns’, are in red. LTR and LC substrings are indicated and correspond to the color coding in Supplemental Figure 1.

Generating 11-2253.ooc file:

blat.exe hg38.2bit /dev/null /dev/null -tileSize=11 –stepSize=5 -makeOoc=11-2253.ooc -repMatch=2253

The ooc file was generated using the guidelines suggested by UCSC [1-2] for using a tile size of 11 and a step size of 5. Using 11-2253.ooc with BLAT masks 11-mers that appear at least 2253 times in the genome.

Identifying unique retroviral vector integration sites (RISs) example:

Supplemental Figure 3 is a simple and hypothetical example of identifying unique RISs. Data from next-generation sequencing can easily result in many unique RISs that only have 1 candidate RIS and some unique RISs that have a frequency of 1,000 or more. At this point, the query sequences have been aligned to the genome, query sequences that could not be aligned to a unique location in the genome have been filtered out, and the candidate RISs have been binned into 10 bp windows. The candidate RIS with the greatest bit score in the 10 bp window is selected as a unique RIS. The span of each RIS in the 10 bp window is derived from the putative integration site to the end of where the query aligns to in the genome. If 2 RISs within a 10 bp window have the same span but a different chromosome start site, these spans are considered distinct.

Example frequency and span count calculation using the unique RIS seq-3:

Frequency = 1 + number of repeat RISs + number of in range RISs = 1 + 3 + 2 = 6

Span count = 5 (distinct bp span values of 180, 182, 189, 190, and 195)

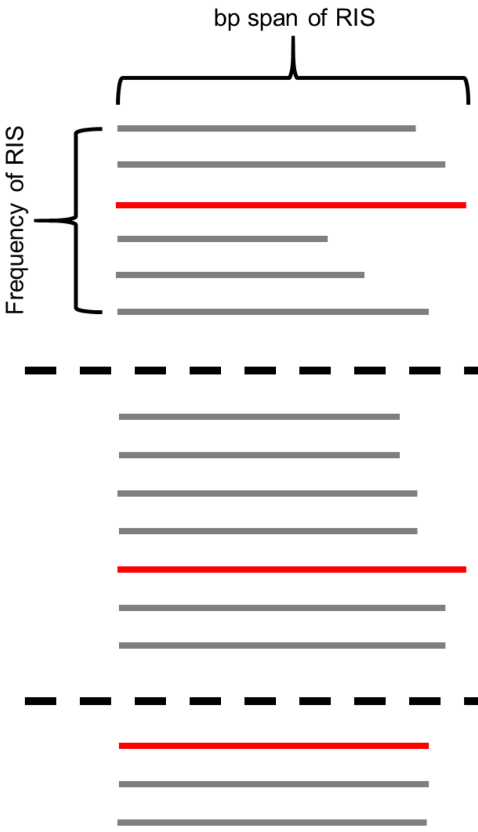

Seq. ID	Chr.	Chr. start site	Chr. end site	Span	Bit score	Designation
seq-1	Chr1	14000000	14000189	190	370	Repeat
seq-2	Chr1	14000000	14000189	190	379	Repeat
seq-3	Chr1	14000000	14000194	195	384	Unique
seq-4	Chr1	14000000	14000179	180	350	Repeat
seq-5	Chr1	14000001	14000182	182	355	In range
seq-6	Chr1	13999999	14000187	189	370	In range
seq-7	Chr1	14200002	14200202	201	390	In range
seq-8	Chr1	14200003	14200203	201	391	In range
seq-9	Chr1	14200001	14200203	203	393	Repeat
seq-10	Chr1	14200002	14200204	203	393	In range
seq-11	Chr1	14200001	14200209	209	397	Unique
seq-12	Chr1	14200001	14200207	207	394	Repeat
seq-13	Chr1	14200001	14200207	207	394	Repeat
seq-14	Chr2	10000001	10000203	203	393	Unique
seq-15	Chr2	10000001	10000203	203	391	Repeat
seq-16	Chr2	10000001	10000203	203	391	Repeat

Supplemental Figure 3 Hypothetical candidate RIS within 10 bp windows. Unique RIS are in red.

A single RIS can be recovered multiple times and mapped to within a few bp of the actual integration site. To account for this, valid RISs should be grouped by an appropriate bp window when determining the unique RISs. To validate grouping RISs into 10 bp windows, we plotted the distribution of aligned sequence reads from an in vivo foamy virus vector study [3]. The ± bp shift was calculated relative to the RIS with the highest capture frequency for all RISs. We observed up to a 5 bp shift in the aligned RIS, suggesting that a 10 bp window (± 5 bp) is appropriate when analyzing large datasets for RISs.

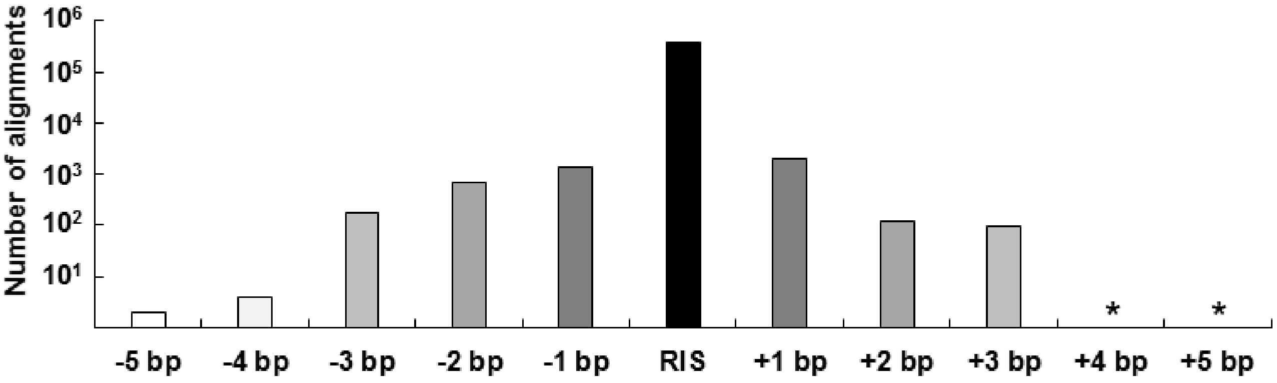

Supplemental Figure 4 Distribution of detected sites. An * indicates a value of 1.

Determining the proximity of unique RISs to a user-provided list of genomic features:

For each unique RIS, VISA determines if it is within a specific genomic feature or its distance to the closest genomic feature. VISA accepts a user-provided list of genomic features that are in the comma separated value (CSV) format. Each row should include the name/description, chromosome, genomic starting location, and genomic ending location of the genomic feature. VISA assumes the values in the first line of the file are the column headers and thus the first line is skipped.

Examining the sensitivity, specificity, accuracy, and false discovery rate (FDR) of VISA:

We generated an *in silico* dataset of 1000 simulated RISs with predetermined genomic locations to analyze the sensitivity, specificity, accuracy and false discovery rate (FDR) of VISA. The dataset was also processed with SeqMap 2.0 [4] and HISAP [5] for comparison, since both of these tools also use BLAT for the alignment process. The advantages of using a simulated dataset are that the chromosome, integration site, and alternative alignments for each RIS can be determined.

Following are the steps we used to generate the simulated dataset: 1) 1000 random locations were chosen from the hg38 build of the human genome. 2) 200 bp were selected upstream starting from each random location to generate the queries. 3) Each query was aligned to the hg38 build of the human genome with BLAT and the ratio of the secondary alignment score and the primary alignment score was calculated (secondary alignment score / primary alignment score = score ratio). 4) Then to simulate RIS sequence reads, a lenti LTR sequence was added to the end of the query corresponding to the randomly generated location and a linker cassette sequence was added to the other end of the query (this file can be downloaded from the VISA website). 5) The predetermined chromosome, integration site, and score ratio were recorded with each query. The latest human genome build supported by HISAP is hg19 and the

latest build supported by SeqMap 2.0 is hg18. Before performing the statistical analyses described below, we used the UCSC liftOver tool to convert the hg38 coordinates used to generate the test dataset to hg19 and hg18 coordinates to account for this.

After processing the test file with VISA, SeqMap 2.0 and HISAP, we analyzed the sensitivity, specificity, accuracy and FDR of each tool. A caveat when performing these analyses is that RISs can have multiple acceptable alignments. One strategy to eliminate RISs that align to multiple locations in the genome is to determine if the ratio of the secondary alignment score and the primary alignment score surpasses a set threshold (alignment similarity ratio threshold). To examine the effects of choosing different criteria, we performed the statistical analyses stated above using alignment similarity ratio thresholds of 0.92, 0.95, and 0.98. Using these criteria, true positives (TP), false positives (FP), true negatives (TN), and false negatives (FN) were designated as follows:

- TP – The query was aligned to the correct chromosome and position (within ± 3 bp) and the score ratio ≤ the alignment similarity ratio threshold.
- FP – The query aligned to an incorrect position, or it aligned to the correct positon but the score ratio > the alignment similarity ratio threshold.
- TN – No integration site was returned for the query and the score ratio > the alignment similarity ratio threshold.
- FN – No integration site was returned for the query and the score ratio ≤ the alignment similarity ratio threshold.
- Sensitivity = TP / (TP + FN), specificity = TN / (TN + FP), accuracy = (TP + TN) / Total, and FDR = FP / (TP + FP)

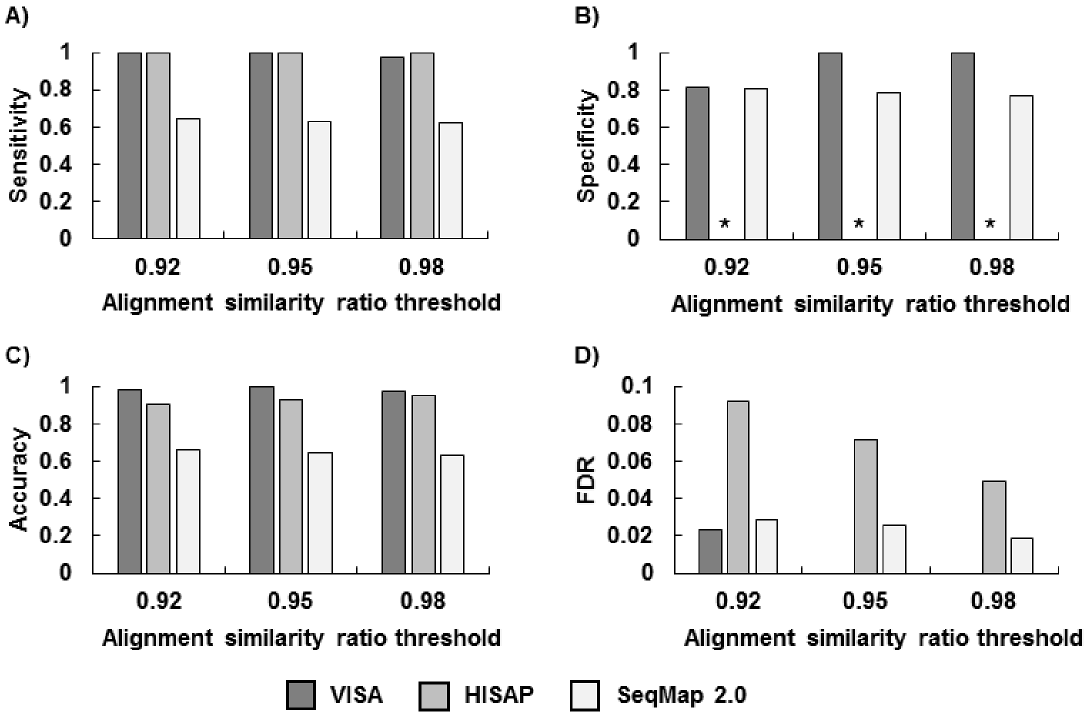

Supplemental Figure 5 A) Sensitivity, B) specificity, C) accuracy, and D) FDR analysis. An * indicates the test is not applicable (HISAP does filter alignments by a score ratio).

Supplemental information references

1. **Blat Suite Program Specifications and User's Guide** [http://genome.ucsc.edu/goldenpath/help/blatSpec.html]

2. **Frequently Asked Questions: Blat** [https://genome.ucsc.edu/FAQ/FAQblat.html#blat6]

3. Olszko ME, Adair JE, Linde I, Rae DT, Trobridge P, Hocum JD, Rawlings DJ, Kiem H-P, Trobridge GD: **Foamy viral vector integration sites in SCID-repopulating cells after MGMTP140K-mediated in vivo selection.** *Gene Ther* 2015.

4. Hawkins TB, Dantzer J, Peters B, Dinauer M, Mockaitis K, Mooney S, Cornetta K: **Identifying viral integration sites using SeqMap 2.0.** *Bioinformatics* 2011, **27**:720–2.

5. Arens A, Appelt J-U, Bartholomae CC, Gabriel R, Paruzynski A, Gustafson D, Cartier N, Aubourg P, Deichmann A, Glimm H, von Kalle C, Schmidt M: **Bioinformatic clonality analysis of next-generation sequencing-derived viral vector integration sites.** *Hum Gene Ther Methods* 2012, **23**:111–8.
